# Perceptions of HIV‐Related Stigma Among Youth Exposed to the PPSAC Programme in Yaoundé, Cameroon

**DOI:** 10.1111/hex.70556

**Published:** 2026-02-14

**Authors:** Bernice Njita, Ivelina Tsocheva, David Hewson

**Affiliations:** ^1^ Institute for Health Research University of Bedfordshire Luton UK

**Keywords:** Cameroon, discrimination, HIV/AIDS, PLWHA, PPSAC, stigma, youth

## Abstract

**Introduction:**

HIV‐related stigma continues to undermine prevention, treatment and quality of life across sub‐Saharan Africa. In Cameroon, stigma persists despite community interventions. This study evaluates the impact of the PPSAC (Projet de Prévention du Sida en Afrique Centrale) project, particularly Result 3, which aims to reduce HIV stigma and discrimination among young people in Yaoundé.

**Method:**

Guided by socio‐cognitive theory, we employed a mixed‐methods design. A total of 260 respondents completed structured online questionnaires, and 24 young people participated in three focus group discussions. Quantitative data were analysed using descriptive statistics and Wilson score 95% confidence intervals. Qualitative data were thematically analysed to explore attitudes, misconceptions and stigma‐reduction strategies.

**Results:**

Findings indicate partial progress in acceptance. While 88.6% of respondents reported willingness to care for an HIV‐positive family member and 57.2% supported HIV‐positive doctors continuing to practise, only 14.7% were willing to befriend someone living with HIV and 67.8% opposed HIV‐positive children attending daycare. Focus group narratives revealed persistent misconceptions about casual transmission, moral and religious condemnation, and family rejection. Conversely, supportive peer networks emerged as protective, fostering resilience and acceptance. Taken together, quantitative and qualitative findings converged to show partial progress in acceptance across several domains, while deeply rooted fears and moral judgements persist.

**Conclusion:**

PPSAC interventions appear to have been only partially successful, with increased acceptance evident in some domains but persistent stigma in others. Future programmes should incorporate family‐ and community‐level engagement, partnership with religious leaders, structural reforms in healthcare and workplaces, and sustained youth‐led advocacy to promote long‐term stigma reduction and improve the well‐being of people living with HIV/AIDS (PLWHA).

## 1 Introduction

The Projet de Prévention du Sida en Afrique Centrale (PPSAC) is a regional initiative aiming to reduce HIV/AIDS stigma and discrimination in Central Africa.

HIV/AIDS remains one of the world's most pressing public health challenges, particularly in sub‐Saharan Africa, which continues to bear the highest global burden [1]. Cameroon has a national HIV prevalence rate of 2.7%, which remains high compared with many other West and Central African countries [2]. Despite decades of prevention campaigns and policy responses, stigma and discrimination towards people living with HIV/AIDS (PLWHA) persist, undermining uptake of testing, adherence to treatment and quality of life [3, 4, 5].

Stigma is recognised as a multifaceted phenomenon encompassing enacted stigma (discrimination), perceived stigma (anticipated negative treatment) and internalised stigma (self‐stigmatisation) [6, 7]. These dynamics exacerbate psychological distress, impede disclosure and hinder access to care [8, 9]. Stigma reduction is therefore essential to achieving the UNAIDS 95‐95‐95 targets for epidemic control [10].

Young people represent a particularly important group for intervention. Adolescents and young adults are at heightened risk of acquiring HIV in sub‐Saharan Africa due to early sexual debut, gender inequities and socio‐economic vulnerability [11, 12]. They are also at a formative stage of attitude development, making them key to shaping future societal norms [13]. However, studies have shown that young people often reproduce stigmatising attitudes prevalent in families, schools and religious communities [14, 15, 16].

However, there has been little systematic evaluation of PPSAC's stigma‐reduction activities in Cameroon, particularly from the perspective of young people. Existing studies on HIV‐related stigma in Cameroon have largely focused on adults or have used single‐method designs, offering limited insight into how youth interpret and respond to stigma‐reduction messages. Mixed‐methods assessments that specifically examine the impact of PPSAC interventions among young people are therefore needed.

In Cameroon, cultural and religious narratives linking HIV to immorality remain powerful [17]. Research has highlighted how HIV is often framed as punishment for sexual transgression, reinforcing blame and silence [18, 19]. Institutional stigma within healthcare settings further compounds these challenges, with PLWHA frequently reporting breaches of confidentiality, neglect or denial of care [20, 21].

Evidence from intervention studies suggests that stigma reduction is most effective when programmes move beyond information dissemination to engage communities, address structural barriers and promote peer‐led advocacy [22, 23, 24, 25]. Within this landscape of multilevel stigma‐reduction approaches, PPSAC represents a major regional initiative that has sought to combine peer education, media campaigns and community mobilisation. The PPSAC project was designed as a regional initiative to address HIV prevention and stigma, with Result 3 focused specifically on reducing stigma and discrimination. In Cameroon, PPSAC activities have included peer education, community mobilisation, youth clubs and media campaigns.

Against this backdrop, there is an urgent need to understand whether current PPSAC activities are effectively reducing HIV‐related stigma among young people in Cameroon. This study addresses this gap by using a mixed‐methods design to assess attitudes and experiences of stigma among youth in Yaoundé who have been exposed to PPSAC interventions. Specifically, we examine patterns of acceptance and discrimination towards PLWHA and explore how young people interpret stigma‐reduction messages, with the aim of informing the design of more contextually grounded and youth‐responsive interventions.

## 2 Method

This study employed a mixed‐methods design, combining quantitative and qualitative approaches to obtain a comprehensive understanding of HIV‐related stigma among young people in Yaoundé. All data collection took place between February and May 2019.

### 2.1 Sampling

Participants were recruited through youth clubs affiliated with PPSAC, university campuses and social media announcements circulated by peer educators. Eligible participants were aged 15–29 years, residing in Yaoundé and able to provide informed consent (or assent with parental consent for those under 18) [15, 16, 17, 18, 19, 20, 21, 22, 23, 24, 25, 26, 27, 28, 29]. Stratified sampling was applied by age group (15–19, 20–24 and 25–29), sex and district of residence to ensure a diverse sample. Equal allocation was used across strata, with approximately the same number of participants recruited within each age category, gender and geographical area to ensure balanced representation. A total of 260 participants completed the questionnaires, and 24 participants took part in the focus group discussions (Table 1).

**Table 1:** Stigma‐related research in sub‐Saharan Africa.

Study	Population	Method(s)	Sample size	Similarity
Li et al. (2011)	Youth	Mixed‐methods	232 survey; 24 FGDs	≈ Our sample
Opuni and Dakwah (2010)	Students	Survey + FGDs	250; 18 FGDs	≈ Our sample
Kalichman and Simbayi (2004)	Adults	Survey	230	Comparable scale
Brown et al. (2001)	N/A	Review	FGDs 6–10	Same FGD model

Sample sizes of comparable magnitude have been reported in stigma‐related research conducted among youth and community populations in sub‐Saharan Africa (e.g., 180–350 survey respondents; 2–5 FGDs with 6–10 participants each). The three FGDs (*n* = 24 participants) were designed to capture diverse perspectives across age and gender; saturation of key themes was observed by the third discussion.

### 2.2 Ethical Considerations

Ethical approval was obtained from the Organization de Coordination pour la lutte contre les Endémies en Afrique Centrale (OCEAC). Written informed consent was obtained from all participants; for participants under 18 years, written assent and parental/guardian consent were obtained. Questionnaires were completed anonymously, and FGD transcripts were de‐identified. All data were stored on password‐protected computers accessible only to the research team.

### 2.3 Data Collection Instruments

Structured questionnaires were used for the quantitative component. These were adapted to align with PPSAC's stigma‐reduction strategies and designed to inform future intervention planning. Data collection was conducted using Google Forms, which enabled efficient and timely responses. Questionnaires were distributed via links sent to participants' mobile phones, requiring internet access to submit validated responses.

To broaden access to the online questionnaire and reduce potential exclusion of youth with limited internet connectivity, the survey link was distributed not only through social media but also via schools, youth organisations and community centres where shared devices were available. These settings enabled participation among individuals who may not have had reliable internet access at home, particularly those in rural or lower‐income communities. Nonetheless, participation remained voluntary and dependent on the local availability of digital resources.

For the qualitative component, FGDs were conducted with one moderator, one note‐taker and one observer. Sessions lasted approximately 90 min and were held in a classroom on campus, selected for participants' convenience. Open‐ended questions were used to explore perceptions of stigma, behavioural change and intervention strategies. The moderator facilitated discussion, encouraged participation and ensured that all views were recorded.

Participants for FGDs were selected purposively from survey respondents who indicated willingness to take part in group discussions, with attention to ensuring variation in age and gender. Each FGD comprised eight participants and was conducted in French. Discussions took place in a quiet classroom on campus and were audio‐recorded with consent, later transcribed verbatim and translated into English. At the start of each session, the moderator emphasised ground rules for confidentiality and respectful discussion.

To enhance credibility, we triangulated quantitative findings with qualitative themes and held regular debriefing meetings among the research team to discuss emerging interpretations and resolve discrepancies.

The full questionnaire and FGD guide are provided as Supplementary File 1.

### 2.4 Data Analysis

Quantitative data were summarised using descriptive statistics. Proportions were calculated for stigma‐related items, with responses grouped into ‘agree’, ‘disagree’ and ‘indifferent/unknown’. Approximate counts were derived by multiplying proportions by the total sample size. Wilson score intervals (95% confidence intervals [CIs]) were then calculated for each proportion and reported in tables. A four‐step process was used to calculate proportions and CIs for stigma‐related items:

1. Proportion estimates—responses were grouped as agree, disagree or indifferent/unknown, and percentages were calculated.
2. Approximate counts—counts (*n*) for each response were obtained by multiplying the proportion by the sample size.
3. CIs—Wilson score 95% CIs were computed for each proportion.
4. Summary tables—results were tabulated to show attitudes towards PLWHA across different measures.

Qualitative data were analysed using inductive thematic analysis. The researcher independently read all transcripts, generated initial codes and developed a coding framework. Transcripts were coded using Excel, and codes were grouped into broader themes such as fear of transmission, moral judgement, religious influence, family rejection, supportive peer networks and institutional discrimination. Representative quotations were selected to illustrate each theme.

### 2.5 Patient and Public Involvement Statement

Young people and community members were involved in several stages of this research. During the design phase, youth representatives and peer educators affiliated with the PPSAC project contributed to refining the study objectives and data collection tools. Their input ensured that questionnaire items and focus group discussion guides were culturally appropriate, understandable and relevant to the lived experiences of young people in Yaoundé.

Participants were not directly involved in data analysis; however, preliminary findings were shared with youth club members and community facilitators through informal dissemination meetings. Their reflections and interpretations helped contextualise the results, particularly regarding the influence of religion, family attitudes and peer support on HIV‐related stigma.

Although participants did not contribute to manuscript preparation, the study was developed with the explicit aim of amplifying their voices and lived experiences. A plain‐language summary of the findings will be shared with youth networks, PPSAC peer educators and community organisations to inform ongoing advocacy and stigma‐reduction activities.

## 3 Results

This study was not designed to evaluate the causal impact of PPSAC, and findings are interpreted descriptively rather than as evidence of programme effects.

### 3.1 Quantitative Findings

Among the 260 respondents, acceptance of PLWHA varied substantially across different social roles and contexts (Tables 2 and 5). Overall, most participants reported willingness to care for HIV‐positive family members, but far fewer were willing to befriend someone living with HIV or support HIV‐positive children attending daycare (Table 3).

**Table 2:** Attitudes towards people living with HIV (all items).

Measure/Item	Agree % (95% CI)	Disagree % (95% CI)	Indifferent/Do not know % (95% CI)
Welcoming PLWHA at home	38.9 (33.1–44.9)	19.4 (14.9–24.5)	41.7 (35.7–47.6)
No change towards the seropositive colleague	37.8 (32.0–43.7)	21.2 (16.6–26.5)	41.0 (35.3–47.2)
Allow PLWHA as service providers	36.3 (30.6–42.2)	30.7 (25.5–36.6)	33.0 (27.6–39.0)
Disgust towards infected homosexuals	30.5 (25.1–36.2)	40.0 (34.2–46.1)	29.5 (22.3–36.0)
HIV‐positive doctors should continue care	57.2 (51.2–63.2)	25.3 (20.5–31.0)	17.5 (13.5–22.8)
The doctor should disclose the patient's HIV status*	—	64.0 (57.8–69.5)	36.0 (30.6–42.2)
Fear of PLWHA (afraid)	—	64.4 (58.2–69.8)**	35.6 (30.2–41.8)**
Would befriend someone with HIV	14.7 (10.8–19.4)	77.7 (72.3–82.3)	7.6 (5.0–11.6)
Would limit contact with PLWHA	23.4 (18.7–29.0)	70.9 (65.0–76.0)	5.7 (3.5–9.3)
Support regardless of infection route	68.2 (62.2–73.5)	21.9 (17.3–27.3)	9.9 (6.7–14.4)

**Table 3:** Summary of respondents' exposure to PPSAC‐related interventions, with 18.2% reporting contact with peer educators and 19% recalling specific media campaigns. The reported channels of sensitisation, with television and social media emerging as the most common sources.

Measure/Item	Agree % (95% CI)	Disagree % (95% CI)	Indifferent/Do not know % (95% CI)
Contact with Reglo Club peer educator (past 12 months)	18.2 (13.9–23.2)	—	81.8 (76.8–86.1)
Heard any ACMS/PPSAC communication campaigns	19.0 (14.6–24.0)	—	81.0 (76.0–85.4)
Knowledge of Reglo Clubs	17.4 (13.2–22.4)	—	82.6 (77.6–86.8)
Television as sensitisation source	45.0 (39.1–51.1)	—	55.0 (48.9–60.9)
Classroom as sensitisation source	31.0 (25.8–37.0)	—	69.0 (63.0–74.2)
Friends/peers as sensitisation source	13.0 (9.5–17.7)	—	87.0 (82.3–90.5)

As shown in Table 2, 88.6% of respondents reported they would care for an HIV‐positive family member, and 71.6% would accept an HIV‐positive family member within the household. However, only 14.7% indicated they would befriend someone living with HIV, and 67.8% opposed allowing HIV‐positive children to attend daycare. Table 5 summarises these patterns, highlighting substantial variation across different domains of social interaction (Table 4).

**Table 4:** Condensed summary of attitudes towards PLWH (percentages).

Measure/Item	Agree %	Disagree %	Indifferent/Do not know %
Accept an HIV‐positive family member	71.6	—	28.4
Accept an HIV‐positive partner	30.7	31.1	38.3
Allow PLWHA as service providers	36.3	30.7	33.0
Buy from an HIV‐positive vendor	70.7	—	29.3
Care for an HIV‐positive family member	88.6	—	11.4
Disgust towards infected homosexuals	30.5	40.0	29.5
The doctor should disclose the patient's HIV status*	—	64.0	36.0
Fear of PLWHA (not afraid)**	64.4	—	35.6
HIV‐positive children should attend daycare	25.0	67.8	7.2
HIV‐positive doctors should continue care	57.2	25.3	17.5
Laws protecting PLWHA should be respected	64.7	21.6	13.7
No change towards the seropositive colleague	37.8	21.2	41.0
Share accommodation with PLWHA	53.7	29.3	17.0
Support regardless of infection route	68.2	21.9	9.9
Welcoming PLWHA at home	38.9	19.4	41.7
Would befriend someone with HIV	14.7	77.7	7.6
Would limit contacts with PLWHA	23.4	70.9	5.7

Key findings summarised across quantitative and qualitative analyses indicate mixed patterns of acceptance. Higher acceptance was observed in family and professional contexts, while stigma remained pronounced in friendship, romantic relationships and childcare settings. Fear of casual transmission and moral and religious judgements were evident across data sources, alongside reports of limited supportive peer environments and ongoing institutional discrimination.

### 3.2 Qualitative Findings

Thematic analysis of FGDs identified six central themes with illustrative quotations (see Table 5).

**Table 5:** Thematic table on HIV Stigma.

Central theme	Sub‐theme	Representative quote	Interpretation/Meaning
Fear of Transmission	Casual contact anxiety	‘I am afraid I can get it by shaking hands or using the same plate’.	Persistent myths about casual transmission indicate gaps in knowledge and limited penetration of educational messaging.
Moral Judgement	Blame and shame	‘People say if you have HIV, it's because you were promiscuous’.	Moral judgements foster social exclusion—especially towards women—and reinforce blame rather than empathy.
Religious Influence	Spiritual condemnation	‘At church, they said HIV is a punishment from God’.	Religious narratives may promote shame and fear, discouraging disclosure and care‐seeking.
Family Rejection	Alienation at home	‘My parents don't allow me to sit with my cousins anymore’.	Familial rejection contributes to isolation, emotional distress and internalised stigma.
Supportive Peer Networks	Youth‐led support	‘My friend told me, “You are still you, nothing changes.”’	Peer support fosters resilience, identity affirmation and emotionally safe spaces.
Healthcare Discrimination	Unequal treatment in care	‘The nurse refused to touch me when she found out I was HIV‐positive’.	Healthcare stigma creates treatment barriers, increases mistrust and worsens health outcomes.
Workplace Stigma	Employment discrimination	‘My boss found out and suddenly I was no longer scheduled for shifts’.	Workplace stigma leads to job insecurity, income loss and fear of disclosure.

### 3.3 Fear of Transmission

Many participants expressed persistent anxieties about contracting HIV through casual contact, reflecting widespread misconceptions about transmission routes. Despite increased exposure to awareness campaigns, fears related to touching, sharing utensils or being physically close to PLWHA remained common. These beliefs shaped participants' willingness to interact with HIV‐positive individuals in public spaces and social settings. Such fear‐driven attitudes were often rooted in inadequate biomedical understanding and reinforced by peer conversations.

One participant explained, ‘I am afraid I can get it by shaking hands or using the same plate’ (FGD2, male, 20). Another added, ‘Even being in the same room makes me nervous because you don't always know how it spreads’ (FGD1, female, 19). These fears often underpinned social distancing behaviours, limiting acceptance even among youth who had participated in PPSAC activities.

### 3.4 Moral Judgement

Moralising attitudes towards PLWHA emerged strongly across discussions. Some participants believed that HIV infection resulted from ‘immoral’ behaviour such as promiscuity or irresponsibility. These perceptions shaped how HIV‐positive individuals were evaluated and often led participants to assign blame rather than express empathy. Such moral judgements appeared to be deeply embedded in cultural norms and reinforced by peer and community discourse.

As one participant noted, ‘People think you must have done something wrong to get HIV, so they judge you before anything else’ (FGD1, female, 22). Another commented, ‘Some youth believe HIV comes from bad behaviour, so they don't feel sorry for the person’ (FGD3, male, 18). These narratives influenced whether participants felt comfortable forming friendships or social ties with PLWHA.

### 3.5 Religious Influence

Religious teachings and interpretations played a significant role in shaping participants' perceptions of HIV. Some youth noted that their religious communities portrayed HIV as a moral or spiritual punishment, reinforcing stigma and shame. These messages created barriers to acceptance and fuelled fears of social judgement within religious settings.

One participant explained, ‘My church says HIV is punishment for sin, so many people grow up thinking that way’ (FGD3, male, 19). Another echoed this sentiment, stating, ‘If you tell your church group you have HIV, they might start treating you differently or avoid you’ (FGD2, female, 21). Religious narratives thus remained a powerful force influencing youth attitudes, despite efforts by PPSAC and other programmes to promote more compassionate perspectives.

### 3.6 Family Rejection

Fear of stigma extended into the family sphere, where some participants described situations in which relatives distanced themselves from HIV‐positive family members. Concerns about community gossip, loss of social status and fear of blame contributed to silence or avoidance within households. Even when family members did not explicitly reject PLWHA, feelings of shame often led to secrecy and limited emotional support.

One participant shared, ‘Families may hide someone who has HIV because they don't want neighbours to know’ (FGD1, female, 18). Another observed, ‘Some parents will not let their children eat with someone who is HIV‐positive because they don't trust what they hear about transmission’ (FGD3, male, 23). These dynamics illustrate how family‐based stigma persists despite increasing awareness in broader community settings.

### 3.7 Supportive Peer Networks

While negative attitudes were common, several participants described peer groups that offered emotional support and displayed inclusive attitudes towards PLWHA. These environments were often linked to prior engagement with sensitisation programmes or personal experiences with HIV in their social circles. Supportive peers helped challenge misconceptions, model acceptance and create safe spaces where youth could discuss HIV‐related concerns.

One participant stated, ‘Among my friends, we talk openly about HIV and nobody is judged—we try to understand more’ (FGD3, female, 24). Another added, ‘My peer group is supportive; we know HIV doesn't spread easily, so we don't treat people differently’ (FGD1, male, 20). These examples highlight the potential of peer influence as a positive driver of stigma reduction and align with socio‐cognitive models emphasising social norms and observational learning.

### 3.8 Healthcare and Workplace Discrimination

Participants reported experiencing or witnessing discriminatory behaviours in institutional settings, particularly in schools, clinics and workplaces. Some students described teachers who treated HIV‐positive learners differently or discouraged their participation in group activities. In healthcare settings, youth noted judgemental comments or visible discomfort from staff when handling HIV‐positive patients. These institutional behaviours reinforced community‐level stigma and discouraged openness about HIV status.

One participant shared, ‘If a student is HIV‐positive, some teachers treat them differently or keep their distance’ (FGD2, male, 21). Another described experiences in clinics: ‘Health workers sometimes look at you in a judging way, like they think they know how you got it’ (FGD1, female, 23). These experiences suggest that institutional stigma remains a significant barrier to creating inclusive and supportive environments for youth.

When interpreted through socio‐cognitive theory, survey patterns suggest that PPSAC activities have contributed to increased HIV‐related knowledge and exposure to prosocial norms in close‐family contexts. However, persistent misconceptions revealed in FGDs and negative peer‐based attitudes indicate that changes in knowledge have not fully translated into positive behavioural intentions or confidence to challenge stigma in broader social environments.

## 4 Discussion

Our findings revealed persistent fears of casual HIV transmission, with some participants expressing anxiety about shaking hands, sharing utensils or being in close proximity to PLWHA. These misconceptions have been widely documented in sub‐Saharan Africa, where biomedical misunderstandings continue to shape fear‐based stigma [14, 27]. Consistent with previous research, inaccurate beliefs about everyday transmission remain a central driver of social distancing and reluctance to form social relationships with PLWHA [7, 30].

The mixed‐methods design of this study allows these quantitative patterns to be interpreted alongside the qualitative narratives, providing a more nuanced picture of youth stigma. Triangulation across the two data strands showed strong convergence: the survey‐identified fears of casual HIV transmission were reinforced by qualitative accounts describing anxiety about shaking hands, sharing utensils or being near PLWHA. Similarly, quantitative reluctance to befriend or publicly associate with PLWHA was mirrored in interview narratives, emphasising fear of judgement from peers. Divergence also emerged: while quantitative data indicated relatively high acceptance of HIV‐positive family members, qualitative discussions revealed lingering moral and religious concerns that may not fully surface in structured survey responses. These complementary insights demonstrate how integrating both strands strengthens the interpretation of the findings beyond what either method alone could reveal.

Moral judgements also strongly influenced participants' attitudes, with several youth attributing HIV infection to ‘immoral’ or irresponsible behaviour. This pattern aligns with studies showing that moralising narratives remain a powerful source of stigma in many African settings, reinforcing blame and reducing empathy towards PLWHA [3, 31]. As previously described in Cameroon and neighbouring contexts, value‐based judgements often prevent individuals from viewing HIV as a chronic medical condition rather than a moral failing [15].

Religious interpretations further contributed to stigma. Some participants described church teachings portraying HIV as divine punishment. This finding is consistent with regional literature showing that faith‐based messages can either mitigate or exacerbate HIV stigma depending on doctrinal framing [12, 29]. While some religious communities promote care and inclusion, others reinforce shame and silence, limiting disclosure and social support [16].

Institutional stigma also emerged, particularly in schools and healthcare settings. Participants described examples of discriminatory behaviour from teachers and health workers, echoing evidence that institutional actors can unintentionally reinforce stigma through avoidance, judgmental language or breaches of confidentiality [18, 32]. Similar findings across Africa show that institutional stigma reduces healthcare uptake and contributes to delays in testing and treatment [5, 20].

Despite these challenges, some participants reported supportive peer networks that openly discussed HIV and promoted acceptance. Peer influence has been shown to be a significant protective factor, with youth‐led spaces often serving as platforms for countering misinformation and modelling non‐stigmatising attitudes [22, 33]. This reflects broader findings that positive peer norms can buffer against community‐level stigma and enhance self‐efficacy to challenge discriminatory behaviour [11].

Finally, structural and social pressures—such as fear of gossip, limited privacy and community expectations—shaped how young people navigated stigma. These contextual factors are consistent with prior studies highlighting the interplay between social norms, household dynamics, and legal or policy gaps that perpetuate stigma in Cameroon and other sub‐Saharan African countries [6, 23]. Without structural shifts in public messaging, institutional training and legal protections, individual behaviour change remains constrained.

Applying socio‐cognitive theory, the findings suggest that PPSAC's influence is strongest at the level of knowledge acquisition and perceived normative expectations within intimate or family environments. High acceptance of HIV‐positive family members likely reflects improved understanding of transmission risk and strengthened descriptive norms in these safe spaces. However, limited willingness to befriend or publicly associate with PLWHA demonstrates that knowledge alone is insufficient to shift broader perceived social norms or enhance self‐efficacy to resist stigma, particularly in peer networks where fear of judgement is salient. Persistent misconceptions about casual transmission and moral or religious interpretations of HIV suggest that outcome expectations and self‐regulatory beliefs remain weak. Thus, PPSAC interventions appear effective in improving what youth know, but less effective in shaping what they believe others expect or what they feel capable of defending in high‐risk social settings.

Finally, structural interventions remain critical. Legal protections for PLWHA exist in Cameroon, but are often poorly enforced. Comparative evidence suggests that stigma is reduced where anti‐discrimination laws are visible and actively upheld [34]. Multilayered approaches that combine education, peer engagement, healthcare reform and legal enforcement are therefore essential for long‐term change [35, 36].

Taken together, the integrated analysis of quantitative and qualitative data highlights the multilayered nature of HIV‐related stigma among youth. Quantitative trends provided a sense of the prevalence of fear‐based, moral and institutional stigma, while qualitative findings explained the mechanisms behind these patterns—including misinformation, religious framing and social pressure.

This study has several limitations. First, attitudes towards PLWHA were self‐reported and may be influenced by social desirability bias, particularly given the sensitivity of HIV‐related topics. Second, the survey was administered online, which likely over‐represents young people with regular internet access and higher educational levels in urban Yaoundé; findings may not generalise to rural or less connected populations. Third, data collection relied on an online questionnaire, which may have limited participation among youth without stable or private internet access. As a result, individuals from rural areas and lower‐income households may be under‐represented in the final sample. This sampling constraint should be considered when interpreting the findings, as it may affect the generalisability of the results. Although efforts were made to distribute the survey through schools and community organisations to increase accessibility, some degree of digital exclusion is likely to have persisted. Fourth, the qualitative component involved a relatively small number of FGDs, and although thematic saturation was reached, some perspectives may not have been captured. Qualitative data were coded primarily by a single researcher, which may have influenced theme interpretation despite efforts at reflexivity.

Future research should include longitudinal evaluations of PPSAC and similar interventions to assess changes in stigma over time and in relation to programme exposure. Studies in rural areas and among out‐of‐school youth are needed to capture a broader range of experiences. In addition, rigorous evaluations of youth‐led and faith‐based stigma‐reduction programmes could help identify which components are most effective in shifting entrenched beliefs and social norms.

## 5 Conclusion

This study demonstrates that although PPSAC activities appear to have contributed to improved knowledge and partial shifts in perceived norms among youth in Yaoundé, substantial HIV‐related stigma persists across personal, social and institutional settings. Interpreted through socio‐cognitive theory, the findings suggest that PPSAC are consistent with partial progress in strengthening knowledge and modestly influencing perceived norms. It appears to have less impact on self‐efficacy and outcome expectations, which are essential for enabling youth to challenge stigma in their wider social environments. Anonymising misconceptions about casual transmission, moral and religious judgements, and discriminatory institutional practices continue to shape attitudes and behaviours.

Strengthening stigma‐reduction efforts will therefore require youth‐centred strategies that combine accurate biomedical information with norm‐challenging activities, peer‐led initiatives and training for teachers, religious leaders and healthcare providers. Future research should examine long‐term changes and explore how community, institutional and policy‐level interventions can better support young people's capacity to resist and challenge HIV‐related stigma.

## Author Contributions

**Bernice Njita:** conceptualisation, methodology, data collection, data analysis, writing – original draft, writing – review and editing, final manuscript approval. **Ivelina Tsocheva:** data interpretation, supervision, writing – review and editing, final manuscript approval. **David Hewson:** writing – review and editing, validation, final manuscript approval.

## Funding

The authors received no specific funding for this work.

## Ethics Statement

Ethical approval for this study was obtained from the OCEAC (Organization de Coordination pour la lutte contre les Endémies en Afrique Centrale). All procedures were conducted in accordance with the ethical standards of the Declaration of Helsinki.

## Consent

All participants provided written informed consent prior to participation. For participants under the age of 18, written assent was obtained alongside parental or guardian consent, in accordance with national and international ethical guidelines for HIV‐related research.

## Conflicts of Interest

The authors declare no conflicts of interest.

## Supporting information

Supporting file 1.

## Data Availability

The datasets generated and/or analysed during the current study are available from the first author on reasonable request.
