# Supplementary material for: Perceptions of HIV‐Related Stigma Among Youth Exposed to the PPSAC Programme in Yaoundé, Cameroon
Source: Health Expect. 2026 Feb 14;29(1):e70556. doi: 10.1111/hex.70556 (PMC12906275; doi:10.1111/hex.70556)
Supplement: Supplementary file 1 — Supporting file 1. [file HEX-29-e70556-s001.docx]

Appendices

Appendix 1: Questionnaire

SECTION 1: Identification of informant

| **S1Q02** | **Sex** 1=Male 2=Female | \|_\| |
| --- | --- | --- |
| **S1Q03** | **Age :** __________________________________________ | \|_\|_\| |
| **S1Q04** | **Marital Status**  1=single 2=free Union 3=Married monogamy  4=Married polygamy 5=widow/widower 6=Separated/divorced | \|__\| |
| **S1Q06** | **Religion**  1=Catholic 2= Protestant 3= Muslim 4=Animist 5= No religion 6= others _____________ | \|__\| |
| **S1Q07** | **Highest Degree**  01=no degree 02=FSLC/CEP 03=BEPC/CAP 04=GCE OL/Probatoire  05=GCE AL /Baccalauréa/BP/BT 06=DEUG/BTS/DUT or equivilent BAC + 2 07=Bachelors or équivalent BAC + 3 08=Maîtrise/Master 1 or equivalent BAC + 4 09=DEA/DESS/Master 2 ou équivalent BAC + 5 10= Doctorat/PhD | \|__\|__\| |

SECTION 2: Behaviour towards PLWHA

I will read some affirmations to you. For every one you will tell me if you

01 = agree 02 = disagree 03= Indifferent . (From S2Q01 to S2Q18).

| **S2Q01** | **My attitude will not change if I learn that my colleague at work is seropositive.** 01 = agree 02 = disagree 03= Indifferent | \|__\| |
| --- | --- | --- |
| **S2Q02** | **PLWHA should have a welcome home of theirs** | \|__\| |
| **S2Q03** | **I can be a friend of someone who has AIDS.** | \|__\| |
| **S2Q04** | 1. **I would limit my contacts with someone who I know is infected with AIDS** | \|__\| |
| **S2Q05** | **My support for someone infected with the AIDS virus depends on how they got infected.** | \|__\| |
| **S2Q06** | **People who are infected in homosexual relationships disgust me.** | \|__\| |
| **S2Q07** | **People who are infected with the AIDS virus should be allowed to serve the public for example as waiter, cook, and hairdresser**. | \|__\| |
| **S2Q08** | **Children infected with the AIDS virus should be able to attend day care**. | \|__\| |
| **S2Q09** | **Doctors with AIDS should be allowed to continue to care for their patients** | \|__\| |
| **S2Q10** | **A doctor should have the right to warn the sexual partners of a person who is infected with the AIDS virus if the latter refuses to do so** | \|__\| |
| **S2Q11** | **If I have a roommate and learn that he is infected with the AIDS virus, I would not mind** | \|__\| |
| **S2Q12** | **People infected with the AIDS virus have to blame themselves.** | \|__\| |
| **S2Q13** | **I can be a friend of someone who has AIDS.** | \|__\| |
| **S2Q14** | **The law put in place for the respect of HIV patient are always violated** | \|__\| |
| **S2Q15** | **Doctors should tell the government the names of people infected with the AIDS virus.** | \|__\| |
| **SECTION 3; Acceptability of persons living with HIV/AIDS**  I will read some affirmations to you. For every one you will tell me if you  **01= yes 02= no 03=don’t know** | | |
| **S3Q01** | **Do you know or personally know (family, friends, colleagues ...) one or more people infected by the AIDS virus**? | \|__\| |
| **S3Q02** | **If a member in your family contacts the HIV virus , would you want their status to be revealed** |  |
| **S3Q03** | **If a member in your family has HIV would you be ready to take care of them** |  |
| **S3Q04** | **Would you buy aliment from a person you know is zero positive** |  |
| **S3Q05** | **If your teacher was HIV positive ,should she be authorized to teach in class** |  |
| **S3Q06** | **Would you accept your partner knowing that they are HIV positive?** |  |
| **S3Q07** | **Do you think seropositve parsons can have children** |  |

SECTION 4: Exposition to the intervention of ACMS in the question of stigma

I will read some affirmations to you. For every one you will tell me if you

01= yes 02= no 03=don’t know

|  |  |  |
| --- | --- | --- |
| S4Q01 | Have you heard or visited the site [www.reglo](http://www.reglo) during this last months |  |
| S4Q02 | Do you know any reglo club around |  |
| S4Q03 | Have you ever been in contact with a pair educator from a reglo club |  |
| S4Q044 | Have you ever hear of 100% jeune journal/ Facebook 100% JEUNE during this last 12 months? |  |
| S4Q05 | Have you had any message of prevention and ccc during this last 12 months |  |
| S4Q06 | **Which local television channel to you watch must**  01= CRTV  02= CANAL-2  03=EQUINOS  04=STV |  |
| S4Q07 | **Which of this messages vehicle on public sports or TV films have you seen.**  01= je prend les chose a main/ I take things by hand  02= meme seropositive Elle reste ma copine/even seropositive she remains my friend  03= meme seropositive Elle reste mon espouse/even seropositive she remains my wife  04= meme seropositive il colleague/ even seropositive he remain a colleague  05= ne pas divulgue le statu des patients  06= un monde sans infection, sans discrimination et sans décès liés au VIH est désormais possible |  |
|  |  |  |

Q. On a scale of 1to 5 how will you express your satisfaction of these messages?

Very satisfied 1……..2………….3………..4…………….5 not at all satisfied.

Appendix 2: Focus Group Discussion Guide

Focus Group Discussion Guide: Perceptions of Strategies to Reduce Stigmatization

1. Access to Information
   1. What information have you received about stigmatization?
   2. How did you first learn about it?
2. Types of Strategies
   1. What types of strategies or materials have you seen (e.g., posters, radio messages, films, community talks)?
   2. Which of these caught your attention the most? Why?
3. Content of Messages
   1. What messages have you noticed that talk about stigma or discrimination?
   2. How did you feel about these messages?
   3. Do you think the messages are clear and relevant for the community?
4. Effectiveness and Influence
   1. Have these messages influenced your attitudes or behaviors in any way?
   2. Do you think the strategies (such as TV, radio, posters) have contributed to reducing stigma and discrimination in your community? How?
5. Channels and Timing
   1. Through which channels (TV, radio, posters, community events, etc.) do you usually receive these messages?
   2. Do you think these channels are effective? Why or why not?
   3. How often do you see or hear these messages? Is the frequency appropriate?
6. Suggestions for Improvement
   1. What other strategies could be added to help reduce stigma and discrimination?
   2. How can existing messages or methods be improved to better reach people in your community?
